# Proteotranscriptomics of ocular adnexal B-cell lymphoma reveals an oncogenic role of alternative splicing and identifies a diagnostic marker

**DOI:** 10.1186/s13046-022-02445-8

**Published:** 2022-07-30

**Authors:** Jiahao Shi, Tianyu Zhu, Huimin Lin, Zhen Liu, Min Zhou, Ziyao Yu, Xiaowen Zhou, Xin Song, Yefei Wang, Renbing Jia, Xianqun Fan, Yixiong Zhou

**Affiliations:** 1 Department of Ophthalmology, Ninth People’s Hospital, Shanghai Jiao Tong University School of Medicine, Shanghai, 200025 P.R. China; 2 Shanghai Key Laboratory of Orbital Diseases and Ocular Oncology, Shanghai, P.R. China; 3 CAS Key Laboratory of Computational Biology, Shanghai Institute of Nutrition and Health, University of Chinese Academy of Sciences, Chinese Academy of Sciences, Shanghai, 200031 P.R. China; 4 Ninth People’s Hospital, Shanghai Jiao Tong University School of Medicine, Shanghai, 20001 P.R. China

**Keywords:** Lymphoma, Ocular adnexal lymphoma, Proteomics, Alternative splicing, ADAR, Biomarker

## Abstract

**Background:**

Ocular adnexal B-cell lymphoma (OABL) is a rare subtype of non-Hodgkin lymphoma. The molecular characteristics of OABL remain poorly understood. We performed an integrated study to investigate the proteotranscriptome landscape and identify novel molecular characteristics and biomarkers of OABL.

**Methods:**

Integrated quantitative proteome and transcriptome were performed on 40 OABL 12 idiopathic orbital inflammation, 6 reactive lymphoid hyperplasia, and 13 aesthetic orbital plastic surgery specimens. Complete clinicopathologic and prognostic data of the patients were recorded.

**Results:**

We identified high global protein-mRNA concordance as a novel characteristic of OABL. High concordance was related to OABL recurrence. By integrated expression profile, motif enrichment and trend analysis, we found that alternative splicing is inflammation-independently dysregulated in OABL. After portraying the aberrant alternative splicing event landscape, we demonstrated the oncogenic role of ADAR, a core splicing regulator that regulates the splicing of Rho GTPase and cell cycle members. We found that ADAR regulates cell proliferation and Rho GTPase inhibitor sensitivity of lymphoma. We identified DNAJC9 as a potential biomarker for OABL in proteomic analyses. Immunohistochemistry and immunofluorescent staining showed the nuclear staining of DNAJC9 was significantly higher in extranodal marginal zone lymphomas compared with inflammation specimens.

**Conclusions:**

These results provide an integrated gene expression profiling and demonstrate that high global protein-mRNA concordance is a prognosis-related molecular characteristic of OABL. We portray the alternative splicing events landscape of OABL, and reveal the oncogenic role of ADAR. We identified strong nuclear staining of DNAJC9 as a promising pathology diagnostic biomarker for extranodal marginal zone lymphomas.

**Supplementary Information:**

The online version contains supplementary material available at 10.1186/s13046-022-02445-8.

## Background

Lymphomas are malignant lymphoid tumors that arise as the clonal proliferation of lymphocytes classified as non-Hodgkin lymphomas (NHLs) and Hodgkin lymphomas. Ocular adnexal lymphoma (OAL) is a rare form of malignant lymphoid proliferation that constitutes 1–2% of NHLs. OALs arise in the conjunctiva, eyelids, and orbit, including the lacrimal gland [1, 2]. Most OALs are B-cell lymphomas. Extranodal marginal zone B-cell lymphoma (EMZL) is the most frequent subtype of OABL (55–69%), followed by diffuse large B-cell lymphoma (DLBCL) (10–15%) [3–6].

While gene expression profiling has led to landmark discoveries of NHLs, few studies have examined ocular adnexal B-cell lymphomas (OABLs) [7–9]. Furthermore, defining the biology of NHLs solely based on the transcriptome is challenging. By combining proteomic and transcriptomic data, proteotranscriptome-based studies have revealed novel insights into the development and progression of malignancies [10, 11], with findings that cannot be revealed by mRNA-based studies. By investigating mass spectrometry (MS)-based TMT labeling quantitative proteome and transcriptome, we provided an integrated gene expression landscape of OABL, revealed the global protein-mRNA concordance as a novel prognostic-related disease characteristic, and identified a novel pathology diagnostic marker.

Our analysis also revealed the importance of the alternative splicing pathway in OABL. It is a posttranscriptional gene regulation approach, which contributes to protein diversity [12]. Dysregulation of alternative splicing has been shown to contribute to the development and progression of various types of malignancy [13]. While some studies have identified mutation of SFs in mantle cell lymphomas (MCLs), alternative splicing in NHLs has not been well studied [14]. We provided a landscape of alternative splicing events (ASEs) as well as their potential biological implication in OABL and further demonstrated the oncogenic nature of the splicing regulator ADAR in OABL.

## Methods

### Patient selection and ethical approval

We reviewed our medical records database to identify patients confirmed by surgical biopsy at the Department of Ophthalmology, Ninth People’s Hospital, Shanghai Jiao Tong University School of Medicine from January 2016 to February 2020. The inclusion criteria were as follows: (1) diagnosis of histologically confirmed OABL, idiopathic orbital inflammation (IOI), reactive lymphoid hyperplasia (RLH), and patients who underwent orbital plastic surgery for aesthetic reasons; (2) availability of clinical and laboratory information at the time of diagnosis; and (3) specimen storage at − 80 °C. Clinical data were obtained from medical records. IOI, RLH, and normal specimens were defined as controls. IOIs and RLHs were defined as the “inflammation” in subgroup analysis (Supplementary Table S1).

The study protocol was approved by the institutional review board of Ninth People’s Hospital, Shanghai Jiao Tong University School of Medicine (protocol SH9H-2019-T185–2). Informed consent was obtained from all patients enrolled in the proteomic and transcriptomic analysis. The clinical characteristics of these patients (40 OABL patients and 31 controls) are summarized in Supplementary Table S2.

### Protein sample preparation and sequencing

Proteomic and transcriptomic data were generated from 71 samples from the above-mentioned patients. The pathological sections were reviewed by three pathologists to validate the diagnosis before sequencing. All specimens were stored at − 80 °C until protein and RNA isolation, and sequencing was performed by Beijing CapitalBio Technology Inc.

The experimental steps are described in the Supplementary Methods. Briefly, specimens were lysed using protein extraction buffer (8 M urea,0.1% SDS) containing 1 mM phenylmethylsulfonyl fluoride (Beyotime Biotechnology, China) and protease inhibitor cocktail (Roche, USA). Tandem mass tags TMTpro (Pierce, USA) with different reporter ions (126–131 Da) were applied as isobaric tags for relative quantification and TMT labeling was performed following the manufacturer’s instructions. The MS analysis was conducted using an Q Exactive mass spectrometer (Thermo Scientific, USA). Proteome discoverer software (version 1.4) (Thermo Scientific, USA) was used to perform database searching against the RefSeq database. The results were filtered using the following settings: high confident peptides with a global FDR < 1% based on a target-decoy approach. The proteomic data have been uploaded into the iProX database (https://www.iprox.org); (project ID IPX0004253000).

### RNA sample preparation and sequencing

RNA samples were prepared using TRIzol reagent (Ambion, 15,596–026) following the manufacturer’s protocol. The poly-A containing mRNA molecules were purified from RNA using poly-T oligo-attached magnetic beads. The fragments were reversely transcribed into first strand cDNA using random hexamers, following by second strand cDNA synthesis using DNA polymerase I and RNase H. PCR was used to selectively enrich DNA fragments with adapter molecules on both ends and to amplify the amount of DNA in the library. The library was qualified using the Agilent 2100 bioanalyzer and quantified by Qubit and qPCR. The produced libraries were sequenced on the illumina Novaseq 6000 platform. Reads were aligned to hg38. The RNA-seq data have been deposited in the Gene Expression Omnibus database (https://www.ncbi.nlm.nih.gov/geo) under accession numbers GSE171059 and GSE199517.

### AASE identification and analysis

The AASEs in OABLs were identified using rMATS [15]. All the sequences and annotations used in this analysis were based on GRCh38 genome assembly. An ASE with a ΔInclevel value between the OABLs and controls of more than 5% (|ΔInclevel | < 0.05) and adj *p*-value of < 0.01 was identified as an AASE. The list of annotated splicing factors and regulators was downloaded from the SpliceAid-F database and the study by Nostrand et al. [16, 17] (Supplementary Table S9).

### Differential expression analysis

For transcriptomic data, low-abundance transcripts were removed. For proteomic data, low-abundance proteins and proteins missing in > 20% patients were removed, and K-nearest neighbor imputation method was used to complete values in proteomic data for proteins missing in < 20% patients. Differential expression analysis was performed using limma R package (version: 3.46.0) [18] after normalization. We set |log2(foldchange)| > log2(1.5) and *p*-value < 0.05 as the threshold for transcriptome data, and |log2(foldchange)| > log2(1.2) and p-value < 0.05 as the threshold for proteome data.

### Functional enrichment analysis

The Metascape tool (http://metascape.org) [19] was used to identify biologic genesets of the selected genes. Gene Set Enrichment Analysis (GSEA, https://www.gsea-msigdb.org) [20, 21] and Gene Set Variation Analysis (GSVA) [22] were performed to investigate deregulated genesets between groups. Particular Molecular Signatures Database collections were included in the GSEA and GSVA analyses. Hallmark (H), curated canonical pathways (C2:CP), and gene ontology BP (C5: GO BP) gene set collections were included to identify genesets.

### Cells and cell culture

NHL cell lines were provided by Cell Bank and Stem Cell Bank, Chinese Academy of Sciences. The Raji and SU-DHL-4 lymphoma cell lines were cultured in RPMI1640 (Invitrogen, CA) supplemented with 10% fetal bovine serum (Gibco) and 1% penicillin/streptomycin (Gibco) and maintained at 37 °C in a 5% CO_2_ humidified atmosphere.

### Virus transduction and generation of stable cell lines

Two individual lentiviral vectors containing shRNAs targeting human ADAR (pLKO.1-shADAR#1: CCGGCGGATACTACACCCATCCATTCTCGAGAATGGATGGGTGTAGTATCCGTTTTTG; pLKO.1-shADAR#2: CCGGGCCCACTGTTATCTTCACTTTCTCGAGAAAGTGAAGATAACAGTGGGCTTTTTG) were purchased from Shanghai Genomditech (Shanghai, China). Non-targeting shRNA was used as the control. The lentiviral vectors and packaging vectors were transfected into the 293 T packaging cell line using the PolyJet In Vitro DNA Transfection Reagent (SignaGen). Retroviral vectors were transfected. Targeted cells were infected with lentivirus in the presence of 8 μg/ml polybrene (Sigma).

### Western blot

Cells were lysed in mammalian protein extraction reagent (Pierce). After protein quantification using a bicinchoninic acid protein assay kit (Pierce), 60 μg of total protein was separated by 10% SDS-PAGE under denaturing conditions and transferred to PVDF membranes (Millipore). Membranes were blocked in 5% non-fat milk (Bio-Rad) and then incubated with primary antibodies, followed by incubation with a secondary antibody conjugated with horseradish peroxidase (1:10,000; Amersham Biosciences). Immunoreactive proteins were visualized using the LumiGLO chemiluminescent substrate (Cell Signaling Technology). The primary antibodies used in this study are as follows: b-Actin (1:10,000; Sigma); and ADAR1 (1:1000; Cell Signaling Technology).

### Cell proliferation assay

To assess cell proliferation, a Cell Counting Kit-8 (CCK-8, New Cell & Molecular Biotech, China) was used following the manufacturer’s instructions. Cells were seeded into a 96-well plate at 1 × 10^3^ cells per well with 100 μl medium and cultured at 37 °C with 5% CO_2_. CCK8 solution was added (10 μl per well) and cells were incubated for 3 h before measuring the absorbance at 450 nm.

### Immunohistochemistry (IHC) and immunofluorescent (IF) staining

IHC and IF were performed following standard procedures. Five-micron thick formalin-fixed paraffin embedded (FFPE) human tissue sections were used for the experiments. Stained slides were digitized using the Pannoramic DESK (3D HISTECH) with a 40× objective lens.

For IF, sections were stained with the Djc9 antibody (#NBP1–87903, Novusbio, 1:50) and CD20 antibody (#GB14030, Servicebio, 1:200) following the manufacturer’s instructions. Mean fluorescent intensity (MFI) was quantified using ImageJ (https://imagej.nih.gov/ij/). Colocalization analysis was performed using ImageJ plugin JACoP.

For IHC, sections were stained with the Djc9 antibody (#NBP1–87903, Novusbio, 1:500). Staining intensity score, nuclear staining intensity score, and staining percentage were quantified using ImageJ IHC Profiler plugin. Staining index was calculated as follows: staining intensity score (0–3) × staining percentage (0–100%). Staining intensity score was graded as follows: score 0: negative staining, score 1: weak staining, score 2: moderate staining, score 3: strong staining.

### Definitions

Local recurrence was defined as lymphoma recurrence at the orbital region. Distant recurrence was defined as lymphoma recurrence at an extra orbital site that was not initially involved. Progression-free survival (PFS) was calculated from the date of diagnosis to recurrence, progression, death, or the most recent follow-up. Overall survival (OS) was defined as the time from initial disease diagnosis to death by any cause or until the most recent follow-up. Recurrence-free survival (RFS), local recurrence-free survival (LRFS), and distant recurrence-free survival (DRFS) were calculated from the date of initial treatment to the corresponding recurrence, or until the most recent follow-up.

### Statistical analysis

All statistical tests were two-sided and *P*-values < 0.05 were considered statistically significant. Statistical analyses were performed using the R software developed by the R Development Core Team at R Bioconductor [23] and GraphPad Prism (https://www.graphpad.com/scientificsoftware/*prism*/).

For comparison of continuous variables, we used the Mann–Whitney U test for correlation results as previously mentioned [11], and the Student’s t-test for others. For correlation analysis, we applied the Spearman rank correlation test for global protein-mRNA correlation following previous observations [24], and Pearson’s correlation test for other continuous variables. Survival curves were generated using the Kaplan–Meier method and compared by log-rank test. Cox proportional hazards models were constructed to identify prognostic factors for OABL. Adjusted hazard ratios with 95% confidence intervals were calculated. Logistic regression models and lasso regression models were constructed to identify diagnostic markers for OABL.

## Results

### Integrated proteotranscriptomic landscape of OABL

**Fig. 1:**
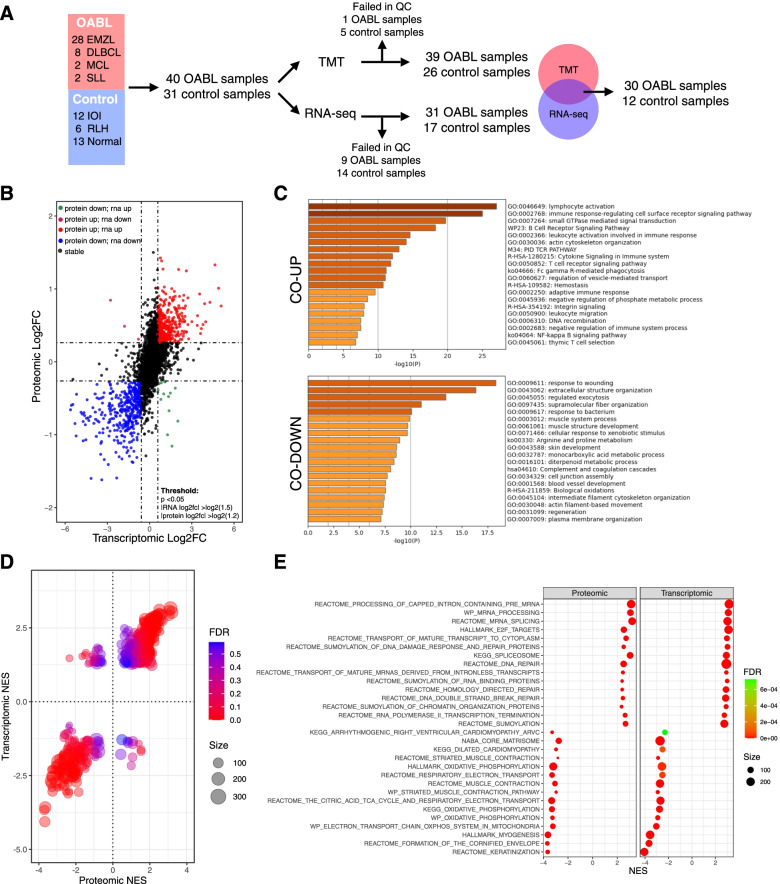
Proteotranscriptomic characteristics of OABL. **A** Sampling workflow of transcriptomic and proteomic cohort. **B** Scatterplot of differentially expressed genes (DEGs) of 3639 protein-mRNA pairs identified in the transcriptomic cohort (31 OABLs and 17 controls) and the proteomic cohort (39 OABLs and 26 controls) between OABLs and controls. The horizontal line is at proteomic |log2(FC)| = log2(1.2); vertical line is at transcriptomic |log2(FC)| = log2(1.5). Genes with *p* ≥ 0.05 are labeled as black. **C** Bar plot of top 20 enrichment terms identified in concordant upregulated and downregulated genes. **D** Bubble plot of differentially enriched gene sets identified by GSEA in the transcriptomic cohort (31 OABLs and 17 controls) and the proteomic cohort (39 OABLs and 26 controls) between OABLs and controls. Gene sets with FDR < 0.2 in at least one of transcriptomic and proteomic analysis are displayed. **E** Bubble plot of top 15 up/down regulated gene sets identified by GSEA

To obtain a comprehensive view of the molecular characteristics of OABLs, we performed an integrated proteomic and transcriptomic analysis of 40 OABLs, including 28 EMZLs, 8 DLBCLs, 2 MCLs, and 2 small lymphocytic lymphomas (SLLs), and 31 control specimens including 12 IOIs, 6 RLHs, and 13 normal orbital tissues (Supplementary Table S1, Fig. 1A). Because of the contamination of plasma proteins, TMT labeled liquid chromatography-mass spectrometry was not performed in one EMZL, three IOI, and two normal samples. Therefore, after a quality control process, we obtained proteomic data of 39 OABLs and 26 controls (Supplementary Table S2). Because of RNA degradation, RNA-seq was not performed in 7 EMZLs, 1 DLBCL, 1 SLL, 4 IOIs, 3 RLHs, and 7 normal tissues. After quality control, we obtained transcriptomic data of 31 OABLs and 17 controls (Supplementary Table S3).

By performing hierarchical clustering of highly variable genes (HVGs) and samples in transcriptomic and proteomic data, we found that most of the OABLs and controls were divided into two groups. While OABL and normal specimens were clearly divided, some of the inflammation samples, especially RLH samples, were misclassified into the OABL group (Supplementary Fig. S1). These results validated our sampling and analysis processes and echoed the observed association between inflammation and lymphoma [25–28].

After removal of low-abundance transcripts/proteins and completion of missing values, we mapped transcripts and proteins to Ensembl IDs and detected 3639 protein-mRNA pairs in these samples. For these pairs, we calculated differentially expressed genes (DEGs) between OABLs and controls (Fig. 1B, Supplementary Table S4). The results showed that 787 genes were upregulated and 542 genes were downregulated in the proteome of OABLs compared with controls. Eight hundred six genes were upregulated and 445 genes were downregulated in the transcriptome of OABLs compared with controls. After matching results of omics data, we found that 451 genes were concordantly upregulated (CO-UP), 386 genes were concordantly downregulated (CO-DOWN), and 19 genes were discordantly dysregulated. The CO-UP DEGs were mainly enriched in immune-related, GTPase signaling, negative regulation of phosphate metabolic process and DNA recombination terms. The CO-DOWN DEGs were mainly enriched in normal tissue development and organization, monocarboxylic acid, and arginine and proline metabolism terms (Fig. 1C).

To avoid reported potential systematic biases [10], we further performed GSEA to identify overlapping dysregulated gene sets in OABLs. A total of 1023 gene sets were commonly identified by proteomic and transcriptomic data (Supplementary Table S5). Among these, 763 gene sets were significantly dysregulated in at least one type of omics (FDR < 0.2), and 725 were concordantly dysregulated and 38 were discordantly regulated (Fig. 1D). Arranged by the sum of NES rank in each omic, CO-UP gene sets were mainly enriched in mRNA processing and splicing, DNA damage and repair, and protein sumoylation. CO-DOWN gene sets were mainly enriched in normal tissue development and organization, and aerobic glucose metabolism (Fig. 1E). As our study contained multiple subtypes of OABL, we performed GSVA in the proteomic data and examined the robustness of the dysregulations (Supplementary Fig. S2A, Supplementary Table S6, Supplementary Methods). The variations of these top-ranked genesets were consistent among all four subtypes. For discordantly dysregulated genes and pathways, immune, Golgi traffic and amide metabolism related gene sets were identified by DEG enrichment and GSEA analyses (Supplementary Fig. S2B, C).

### Global protein-mRNA concordance is a distant recurrence-related characteristic of OABL

**Fig. 2:**
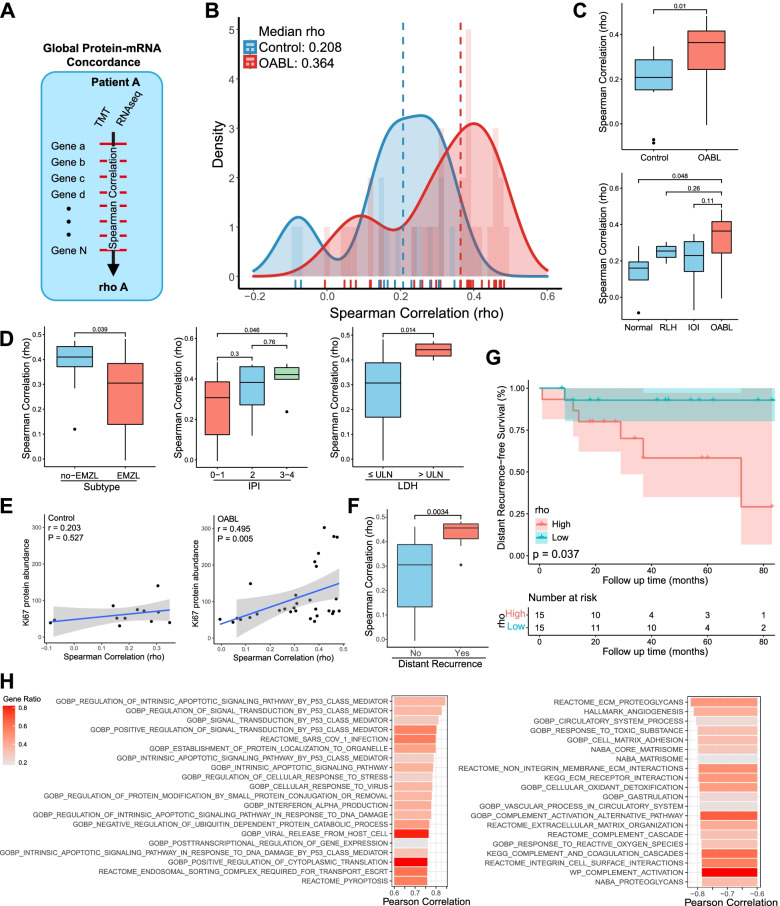
The match-subject analysis identifies global protein-mRNA concordance as an OABL-associated characteristic. **A** Schematic diagram of global protein-mRNA concordance calculation. **B** Density plot showing the global Spearman correlation for protein-mRNA pairs within OABLs (*n* = 3818 protein-mRNA pairs) and controls (*n* = 3728 pairs). **C** Concordance of protein-mRNA pairs is significantly higher in OABLs compared with the control or normal group and relatively higher compared with the RLP or IOI group. **D** Global protein-mRNA concordance is associated with prognostic risk factors. No-EMZL subtype, high LDH, and high IPI score have an increased concordance. **E** Global protein-mRNA concordance is positively correlated with the MKI67 proteomic abundance in the OABLs (r = 0.495, *p* = 0.005) but not in the controls (r = 0.203, *p* = 0.527). Blue line shows liner regression. **F** High global protein-mRNA concordance is associated with distant recurrence in OABL. **G** Kaplan-Meier plot shows high global concordance in OABLs is associated with decreased distant recurrence-free survival. **H** Bar plot of top 20 gene sets identified by GSVA correlated with global protein-mRNA concordance

Next, we examined the relationship between protein and mRNA abundance and its association with disease characteristics. Global protein-mRNA concordance was computed as the Spearman correlation result of all paired protein and mRNA abundance in each patient (Fig. 2A). We analyzed this concordance in patients with both proteomic and transcriptomic data (Fig. 1A). Considering the different transcript/protein abundance distribution between OABLs and controls, we analyzed protein-mRNA pairs separately in these two groups. We identified 3818 protein-mRNA pairs in OABLs and 3728 pairs in controls. The concordance was significantly higher in OABLs (median rho = 0.364) than in controls (median rho = 0.208, *p* = 0.01, Fig. 2B-C). Considering the association between inflammation and lymphoma [25–28], we compared the global concordance across subgroup specimens. The results showed that OABLs exhibited a relatively higher concordance than other groups (median rho of RLH = 0.254, IOI = 0.23, normal = 0.16). These data indicated that the increased correlation between protein and mRNA abundance is a disease-associated characteristic of OABL.

We next analyzed if the concordance is associated with disease aggressiveness. First, we compared the concordance across subtypes, Ann Arbor stage, and prognostic risk factors (Fig. 2D). The concordance within no-EMZL subtypes was significantly higher than that of EMZL (*p* = 0.039), an indolent lymphoma subtype. High concordance was significantly associated with higher LDH (*p* = 0.014) and IPI (*p* = 0.046), two prognostic risk factors of NHL. High concordance was relatively associated with a higher Ann Arbor stage (*p* = 0.054) (Supplementary Fig. S3A). Next, we evaluated the correlation between proliferation ability and global concordance (Fig. 2E). The result showed a strong positive association between Ki67 protein abundance and global concordance in OABLs (r = 0.495, *p* = 0.005), but this association was not present in the controls (r = 0.203, *p* = 0.527). Hence, higher global concordance was associated with disease aggressiveness solely in OABL.

We then tested the correlation between the protein-mRNA concordance and OABL prognosis. We divided OABL patients into two groups using the median value of concordance and compared PFS, OS, RFS, LRFS, and DRFS between the two groups (Fig. 2G, Supplementary Fig. S3B). Among the 15 patients in the high rho group, 6 showed recurrence, 1 showed local recurrence, and 6 showed distant recurrence. For the 15 patients in the low rho group, 2 showed recurrence, 1 showed local recurrence, and 1 showed distant recurrence (Supplementary Table S1). Survival analysis revealed that a globally increased concordance in OABL was significantly associated with reduced DRFS (*p* = 0.037) and relatively associated with reduced RFS (*p* = 0.083), but not with PFS (*p* = 0.19) or OS (*p* = 0.26).

We then compared the global concordance between patients with and without the recurrence events (Fig. 2F, Supplementary Fig. S3C). High concordance was significantly associated with distant recurrence events (*p* = 0.0034) and recurrence events (*p* = 0.0072), but not with local recurrence events (*p* = 0.41). We analyzed the relationship between the global concordance and recurrence in small B-cell lymphoma (SBL), EMZL, DLBCL, and other subtypes to ensure the robustness of the finding (Supplementary Fig. S3C). High concordance was significantly associated with distant recurrence events in SBL (*p* = 0.0059) and EMZL (*p* = 0.039). Despite the low incidence of recurrence and limited number of patients, the median value of patients with distant recurrence was still higher than that of patients without the events in DLBCL and other subtypes. These data demonstrated that the high global protein-mRNA concordance was a predictive factor for distant recurrence in OABL.

Next, we investigated the potential regulators and biological implications of the abnormally upregulated global protein-mRNA concordance. Because the global concordance is an intrinsic continuous variable, we examined the correlation between GSVA results and the concordance (Fig. 2H). In the top 20 positively correlated genesets, 8 gene sets were TP53-related gene sets, and the others were mostly immune-related gene sets. ECM-associated gene sets accounted for the majority of negatively correlated gene sets.

These findings indicate that increased global protein-mRNA concordance is a novel molecular characteristic of OABL that is associated with disease aggressiveness and higher risk of recurrence. This abnormally upregulated concordance in OABL is positively related to the TP53 pathway.

### Trend analyses identify alternative splicing as an inflammation-independent signature of OABL

**Fig. 3:**
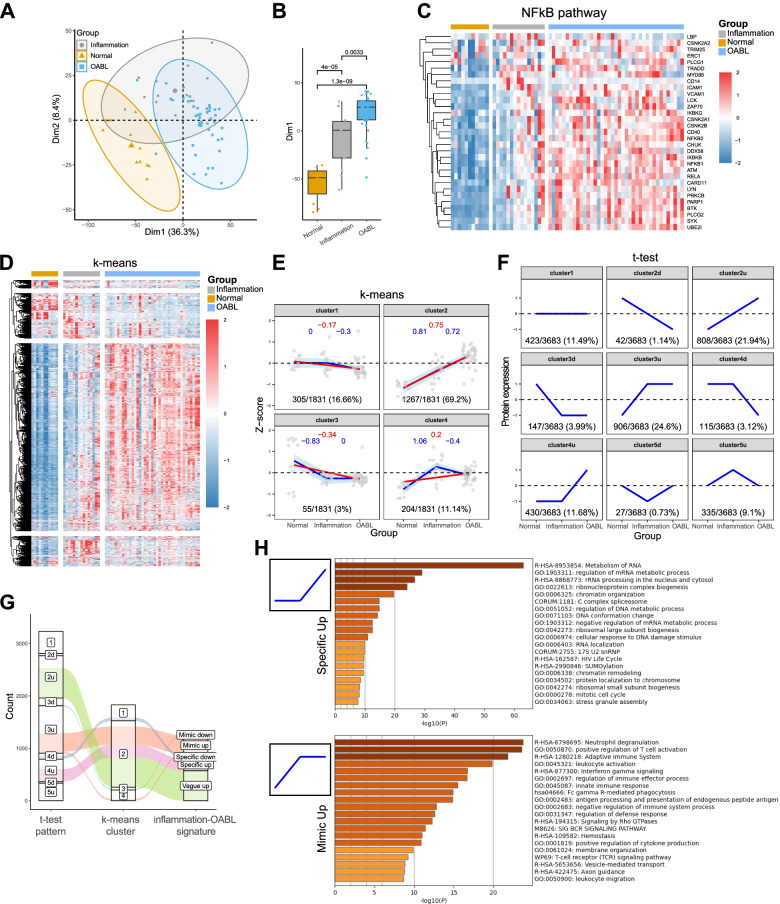
Establishment of inflammation-OABL protein signature. **A** PCA of high variant proteins (protein with top 25% median absolute deviation). **B** boxplot of Dim1 showing difference between normal, inflammation, and OABL groups. **C** Heatmap of NFkB pathway analyzed by proteomic data. **D** Heatmap of k-means clustering result of highly variant proteins (protein with top 50% median absolute deviation). **E** Facet plot showing the trend of protein abundance of each k-means cluster between groups. Each dot represents the median protein z-score for one sample in each cluster. The blue lines indicate the segmented linear regression between two adjacent groups, and the blue numbers indicate the slope value of the regression. The red lines indicate linear regression for all groups, and the red numbers indicate the slope value of the regression. The numbers in black indicate the percentage of genes in the cluster compared to all genes analyzed. **F** Facet plot shows nine patterns of protein expression changes across groups identified by t-test. Black numbers indicate the percentage of genes in the cluster compared to all genes analyzed. **G** Alluvial plot showing the process of inflammation-OABL protein signature establishment. **H** Bar plot of top 20 enrichment terms of Specific up signature and Mimic up signature

In the proteotranscriptomic data, we observed a similarity of molecular characteristics between inflammation and OABL samples through hierarchical clustering, principle component analysis, and global protein-mRNA concordance (Fig. 2C, Fig. 3A-B, Supplementary Fig. 1). As previous studies demonstrated the activation of NFκB signaling pathway in both inflammation and NHL [25, 26], we performed hierarchical clustering in the NFκB signaling pathway across subgroups (Fig. 3C). The abundance of NFκB-related genes progressively increased from normals to inflammations, and to OABLs, which was consistent with the previous reports [25, 26]. However, issues remained as: what extent the similarity is; which pathways discriminate OABL from inflammation; and whether these pathways are driver events of OABL.

To address these questions, we constructed a robust inflammation-OABL signature in proteomic data by supervised and unsupervised clustering genes across the normal, inflammation, and OABL groups. First, we performed the t-test of protein abundance between each two groups and hypothesized nine dysregulated patterns of genes (Fig. 3F, Supplementary Methods). Most were constituted by the upregulated patterns (cluster3u 24.6%; cluster2u, 21.94%; cluster4u, 11.68%). Interestingly, in these upregulated patterns, inflammations could not be discriminated from OABLs in a majority of genes (906/2144, 42.3%). We additionally performed an unsupervised k-means clustering for normalized HVGs (genes with top 50% MAD, k = 4) (Fig. 3D-E). Cluster numbers were determined by the elbow plot (Supplementary Fig. S4A).

Combining the results of k-means clustering and t-test gene patterns, we identified five clusters of genes: inflammation mimic upregulated genes (MIMIC-UP), vaguely upregulated genes (VAGUE-UP), OABL specific upregulated genes (SPECIFIC-UP), inflammation mimics downregulated genes (MIMIC-DOWN), and OABL specific downregulated genes (SPECIFIC-DOWN) (Fig. 3G, Supplementary Table S7). Because upregulated genes constituted most of the clustered proteins, we focused on MIMIC-UP and VAGUE-UP, which represented extremely different patterns of dysregulation. MIMIC-UPs were mostly enriched in immune-related gene sets, while SPECIFIC-UPs were mostly enriched in gene sets that related to mRNA metabolism and splicing, DNA damage and metabolism, and chromatin remodeling (Fig. 3H).

These results clearly demonstrated that the similarity between inflammation and OABL is not only in the NFκB pathway but also in a larger immune landscape. More importantly, we identified gene sets specifically dysregulated in OABL, including mRNA splicing and well-known pathways associated with malignancy development (DNA damage, chromatin remodeling).

### Alternative splicing and its regulators potentially influence OABL development and progression

Alternative splicing plays an important role in OABL. Our findings revealed dysregulated alternative splicing in an inflammation-independent pattern in OABLs (Fig. 1E, Fig. 3H). The mRNA splicing geneset was upregulated in all subtypes of OABL (Supplementary Fig. S5A). We further investigated the enriched motif/domain of OABL in the proteome, and the RNA recognition motif was the most significantly enriched term (Supplementary Fig. S5B). Alternative splicing was reported to be associated with malignancy development and progression [29, 30]. Therefore, we hypothesized that alternative splicing may play an oncogenic role in OABL.

**Fig. 4:**
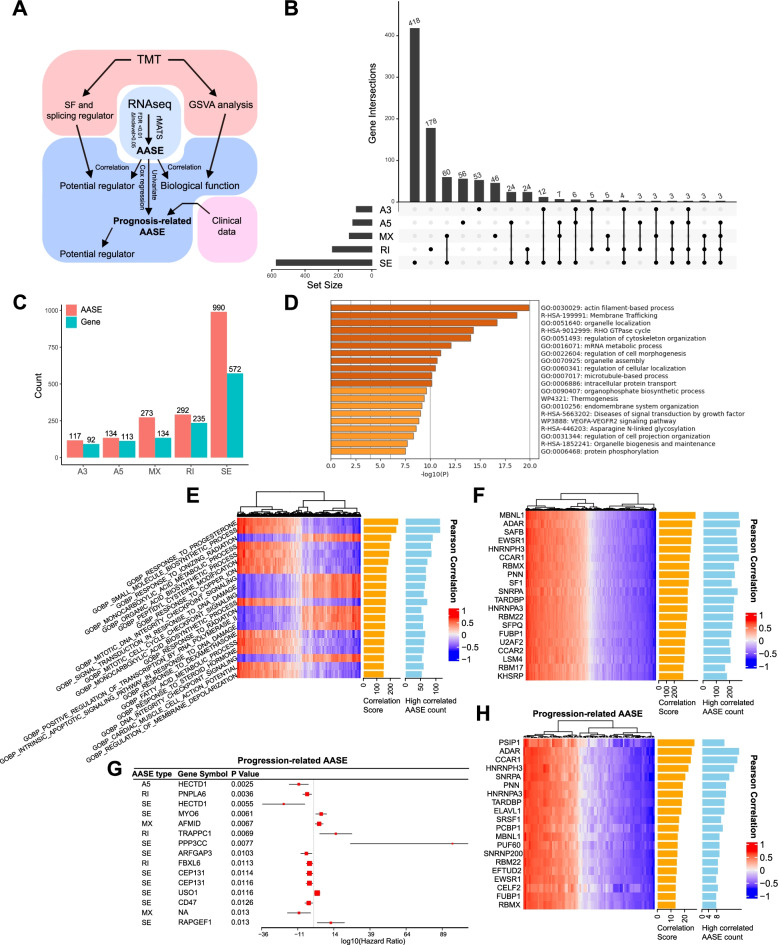
Integrated aberrant alternative splicing event (AASE) landscape of OABL. **A** Workflow of AASE analysis. **B** UpSet plot showing intersections among the five types of AASEs in each patient. **C** Bar plot showing the number of the five types of AASEs and related genes. **D** Bar plot of top 20 enrichment terms identified in AASE-related genes. **E** Heatmap of top 20 enrichment terms correlated with Inclevel of AASEs identified by GSVA. Enrichment terms are ranked by the correlation score and shown in the orange bar. Count of high correlated AASEs with |r| > 0.6 is showed in blue bar. **F** Heatmap of top 20 splicing factors and regulators correlated with Inclevel of AASEs. **G** Forrest plot of top 15 progression-related AASEs ranked by *p*-value. Progression-related AASEs are calculated by cox regression. **H** Heatmap of top 20 splicing factors and regulators correlated with Inclevel of progression-related AASEs

We therefore constructed a workflow to 1) evaluate raw RNA-sequencing data and identify AASEs of OABLs compared with controls; 2) syndicate clinical data to identify prognostic-related AASEs; and 3) combine proteomic data to investigate potential splicing regulators and biological function of AASEs (Fig. 4A). We analyzed five types of ASEs: alternative 3′ splice sites (A3), alternative 5′ splice sites (A5), mutually exclusive exons (MX), retained introns (RI), and skipping exons (SE). A total of 1806 AASEs were identified (Supplementary Table S8), and most were SE. These AASEs affected 916 genes in total, and most of them were affected by SE (Fig. 4C). Among the 916 AASE related genes, 651 genes were only modulated by one type of ASE, while the rest were affected by several types of ASE (Fig. 4B). Interestingly, 60 of 134 MX related genes were also affected by SE. Using univariate cox regression analysis, we identified 91 progression-related AASEs (64 affected genes), including 59 SE, 15 MX, 8 RI, 7 A5, and 2 A3 (Fig. 4G, Supplementary Table S10).

Next, we investigated the biological implication of the AASEs. The top listed enrichment terms of the AASE related genes were associated with the gene sets related to cytoskeleton, Rho GTPase, and organelle (Fig. 4D). Noticeably, Rho GTPase and cytoskeleton pathway were also identified in the enrichment analysis of CO-UP and CO-DOWN DEGs (Fig. 1C). We then analyzed the potential implicated biological function through correlating AASE Inclevel with GSVA results from proteomic data (Fig. 4E, Supplementary Methods). Among the top 20 listed genesets, 8 were related to DNA damage and cell cycle. Others most included steroid hormone-related and organic acid-related gene sets.

We then investigated the potential regulators of AASEs. We correlated the protein abundance of annotated SFs with Inclevel of AASEs (Fig. 4F, Supplementary Methods) and progression-related AASE (Fig. 4H). Among the top 20 listed SFs, 13 were recurrently identified in both correlations. We searched these 13 genes in 4 DLBCL cohorts in cbioportal (http://cbioportal.org) [31]. While 4 genes exhibited no genomic events. While a total of 10% (30/300) patients had genomic events in the other 9 genes (Fig. S5C).

Together these data indicate that AASEs are widely present in OABLs and associated with prognosis. Genes that exhibited alternative splicing were associated with dysregulated gene sets in OABLs. AASEs were associated with key biological functions, like DNA damage and cell cycle, which might imply that they function as post-transcriptional regulators of these process. Some DLBCL patients had genomic events in AASEs that were highly correlated splicing factors and regulators, which also suggested that alternative splicing was a driver event of OABL.

### ADAR is a core regulator of alternative splicing in OABL and influences key biological functions

ADAR, a member of the adenosine deaminases acting on the RNA family of enzymes, catalyzes the editing of adenosine to inosine in double-stranded RNA. ADAR was recently reported to regulate alternative splicing independent of editing ability [32]. In correlation analyses, we found that ADAR was a top-listed SF recurrently associated with AASEs and progression-related AASEs (Fig. 4F, H). Among the SFs recurrently associated with AASEs, ADAR showed the highest incidence of genomic events (3%, 9 of 300 patients).

**Fig. 5:**
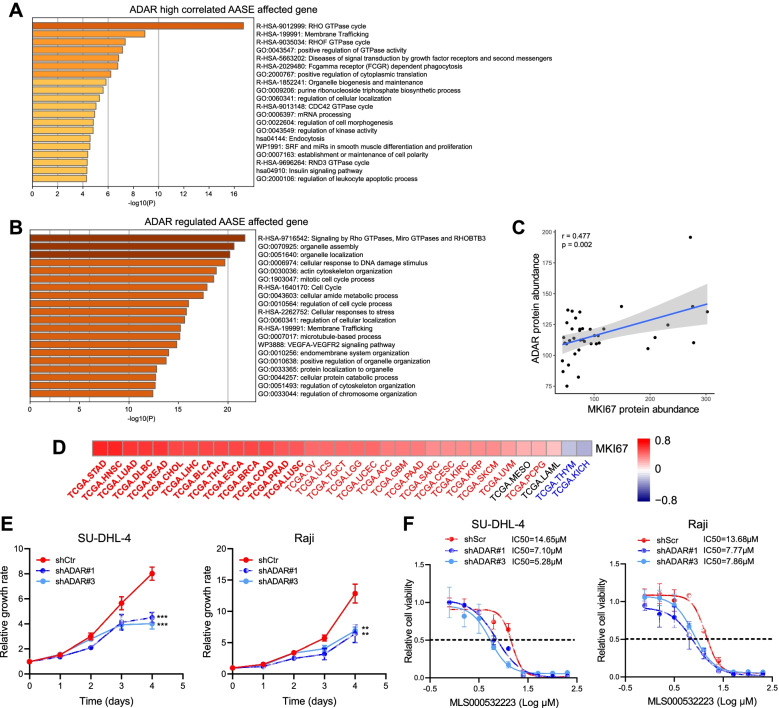
ADAR regulates key biological functions in OABL. **A** Bar plot of top 20 enrichment terms identified in ADAR highly correlated AASE genes (IrI > 0.6, *p* < 0.05). **B** Bar plot of top 20 enrichment terms identified in ADAR regulated AASE affected genes (frequency ≥ 2). **C** ADAR protein abundance is significantly positively correlated with MKI67 abundance in OABL. Blue line shows linear regression. **D** ADAR RNA expression level is significantly positively correlated with MKI67 level in most TCGA databases. Name of databases labeled with red/blue color if *p* < 0.05. Name of databases is bold if |r| > 0.4. **E** ADAR KD SU-DHL-4 and Raji exhibit decreased cell proliferation ability. **F** SU-DHL-4 and Raji cells with control ADAR knockdown are treated by Rho GTPase inhibitor MLS000532223 for 72 hours. ADAR knockdown cell lines exhibit lower IC50

We next investigated the potential biological functions associated with ADAR and found that, the protein abundance of ADAR was strongly correlated with 280 AASEs (|r| > 0.6), which affected 145 genes. These genes were mostly enriched in Rho GTPase-related gene sets (Fig. 5A), an important geneset and critical transducer of intracellular signaling in tumor initiation and progression [33, 34].

To examine the role of ADAR, we identified AASE in 11 groups of ADAR knockdown (KD), knockout, or overexpression cancer cell lines (Supplementary Methods). A total of 1472 genes were recurrently affected by ADAR regulated AASE (Supplementary Table S11). These genes were also enriched in Rho GTPase-related gene sets (Fig. 5B). Because the affected genes were enriched in apoptotic and proliferation related gene sets (Fig. 5A-B), we investigated the relationship between ADAR and MKI67. In our data, ADAR protein abundance was positively correlated with MKI67 (r = 0.477, *p* = 0.002, Fig. 5C). Among 33 tumor types in TCGA databases, 29 exhibited a significantly positive correlation with ADAR expression (*p* < 0.05, r > 0) and 14 exhibited a strong correlation with ADAR expression (*p* < 0.05, r > 0.4) (Fig. 5D).

To verify the hypothetic regulator role, we established ADAR KD NHL cell lines (Fig. S6A). ADAR KD cell lines exhibited significantly decreased cell proliferation compared with the control cells (Fig. 5E). We further found that ADAR KD sensitized NHL cell lines to Rho GTPase inhibitors. The IC50 of the Rho GTPase inhibitor MLS000532223 after 72 h of treatment was lower in ADAR KD cell lines compared with controls (Fig. 5F). ADAR KD cell lines also exhibited increased sensitivity to a Rho-kinase inhibitor (HA110 HCL). These results demonstrated that ADAR, a core regulator of alternative splicing in OABL, regulated cell proliferation and sensitivity to Rho GTPase inhibitors.

### Proteomic analysis identifies DNAJC9 as a diagnostic marker of EMZL

**Fig. 6:**
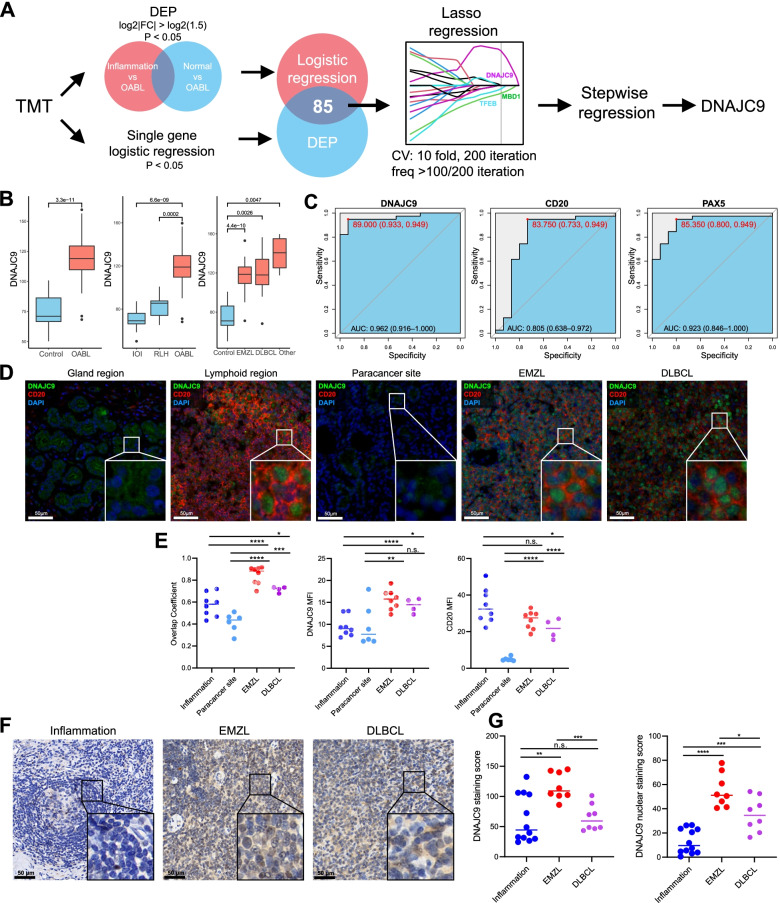
Proteomic analysis identifies DNAJC9 as a diagnostic marker of OABL. **A** Workflow of identifying a diagnostic marker from proteomic data. **B** DNAJC9 protein abundance is significantly higher in OABLs and any subtype of OABL. **C** ROC plot of DNAJC9, CD20, and PAX5. DNAJC9 protein abundance exhibits higher AUC than CD20 and PAX5. **D** Representative IF images of DNAJC9 in control and OABL samples. **E** MFI of DNAJC9, and overlap coefficient of DNAJC9 and DAPI are significantly higher in EMZL and DLBCL samples compared with inflammations and paracancer sites. MFI of CD20 are significantly higher in EMZL and DLBCL samples compared with paracancer sites. Each dot represents a sample. **F** Representative IHC images of DNAJC9 in control and OABL samples. **G** Staining score of bulk cells and nuclei of DNAJC9 in Inflammation, EMZL, and DLBCL samples. Each dot represents a sample

Pathological diagnosis of OABL remains difficult. Therefore, we examined our proteomic data to investigate a potential diagnostic marker for OABL. A workflow of biomarker detection is shown in Fig. 6A. To screen biomarkers, we first identified 98 differentially expressed proteins and 98 significant proteins through univariate logistic regression. Next, 85 overlapped proteins were included into the lasso penalty regression model, and the analysis yielded four proteins (DNAJC9, TFEB, SUMO3 and MBD1) through 200 iterations of cross-validation. We then performed stepwise logistic regression for these proteins, and DNAJC9 was the only protein identified.

To verify the result, we analyzed the protein abundance of DNAJC9 across groups (Fig. 6B). DNAJC9 abundance was significantly higher in OABLs compared with control, IOI, and RLH groups. DNAJC9 abundance was also significantly higher in all subtypes of OABL compared with controls. Compared with the traditional diagnostic markers CD20 and PAX5, DNAJC9 exhibited a relatively higher AUC value for OABLs (Fig. 6C).

Next, we investigated the diagnostic performance of DNAJC9 in patient FFPE samples. EMZL and DLBCL (the two main subtypes of OABL) were chosen as the experimental group, and inflammation samples were used as the control; paracancer sites of OABLs were additionally counted as the control in IF analysis. Analysis of subcellular localization revealed that DNAJC9 was localized in the nucleus in OABLs and lymphoid regions of inflammation tissues. In paracancer sites of OABL and gland regions of inflammation, DNAJC9 was localized in the cytoplasm. Most DNAJC9 was co-expressed with CD20 in the same cell in lymphoid regions and OABLs (Fig. 6D-E). The MFI of DNAJC9 was significantly higher in EMZLs and DLBCLs compared with controls. The MFI of CD20 was not higher in OABLs compared with inflammations (Fig. 6E). IHC analyses showed DNAJC9 staining score of bulk cells and nuclei were both significantly higher in EMZLs compared with inflammations (Fig. 6F-G). For DLBCLs, the staining score of bulk cells in was not significantly higher compared with inflammations, and the staining score of nuclei was significantly lower compared with EMZLs. These results demonstrated that DNAJC9, especially strong nuclear staining of DNAJC9, is a promising pathological diagnostic marker of EMZL that can differentiate EMZL from inflammation.

## Discussion

OABL is a rare subtype of NHL and a common type of malignancy in the orbital region [1, 2, 35]. To date, only a few studies have examined the gene expression profile of OABL [7]. Although there are several transcriptomic studies of NHLs, their proteomics and integrated molecular characteristics have been poorly understood [7–9]. Our study reports proteotranscriptomic data of OABL for the first time. We performed integrated quantitative proteome and transcriptome analyses, that allowed us to: 1) identify robust dysregulated genes and pathways; 2) gain insights into post-transcriptional expression regulation; and 3) investigate novel disease characteristics of OABL. Together, our findings provide novel insights into the molecular landscape of OABL and identify a promising diagnostic biomarker.

In our data, proteome described disease pathways and DEGs are partially captured by the transcriptome. Different distribution between transcriptomic and proteomic data was previously observed [10]. Thus, we additionally performed GSEA, a rank-based algorithm, to investigate the dysregulated pathways in OABL. The robustly concordant dysregulations are mostly consistent with previous observations in mature B-cell lymphomas [9]. Post-transcriptional regulation mechanisms of gene expression, including protein sumoylation, RNA m6A modification, and alternative polyadenylation, are current focuses in cancer research [36, 37]. These processes can result in different expression patterns between protein and mRNA. We assumed that by computing the correlation between transcript and protein abundance, the global protein-mRNA concordance can imply the level of impact of post-transcriptional regulation mechanisms for each patient. Consistent with previous observations in breast cancer, our findings showed that high concordance is a disease-specific characteristic in OABL and associated with poor prognosis [11]. These results suggest that traditional translational regulation of gene expression and expression-independent post-transcriptional modification play a major role in malignancy development.

The similarity and association between inflammation and NHLs are frequently reported, but the extent and underlying mechanisms of this relationship have not been thoroughly studied [25–28]. Herein, we observed a similar situation [25, 26]. We therefore constructed a robust inflammation-OABL signature. NFκB pathway is previously reported upregulated in both inflammation and NHL. In our data, the gene expression profiles of inflammation and OABL are similar and broadly affects immune-related genes, which confirms and extends the scope of this similarity. Echoing our previous assumption, alternative splicing as an expression-independent post-transcriptional regulation mechanism is specifically dysregulated in OABL in an inflammation-independent manner.

Alternative splicing is a post-transcription regulator of pre-mRNA and allows the generation of multiple splice isoforms from genes that can exhibit distinct functions [12]. Numerous studies have demonstrated the oncogenic role of alternative splicing in cancers [29, 30]. Though components of the spliceosome are recurrently mutated in hematologic malignancies [14], including the SF3B1 mutation present in approximately 10% of chronic lymphocytic leukemia and DDX41 mutation in follicular lymphoma and Hodgkin lymphoma, the biological function and oncogenic potential of alternative splicing have not been well studied [38]. From our results on specific dysregulated gene expression and enriched RNA binding motif, we speculate that alternative splicing is a potential oncogenic event in OABL. By constructing an AASE landscape, we found that AASEs are highly correlated with important biological functions, and some AASEs predict the progression of OABL. Our analysis further identified ADAR as a core SF of AASEs. ADAR is recurrently and highly correlated with all AASEs and prognostic-related AASEs and mutated in DLBCL patients. ADAR directly edits and splices RNA and promotes malignancy development and progression [32, 39–42], but its oncogenic role in NHL has not been demonstrated. High correlated AASE affected genes in the OABL and ADAR regulated AASE affected genes identified using publicly available datasets were both enriched in Rho GTPase and cell proliferation pathways. We further showed that ADAR regulates cell proliferation and sensitivity to Rho GTPase inhibitors in NHL cell lines. Together, our findings indicate that alternative splicing is an inflammation-independent oncogenic event of OABL, and dysregulation of the splicing regulator ADAR may result in malignancy development and progression.

A major issue in clinical practice is the efficient clinical or pathological differential diagnosis between OABL (especially EMZL) and inflammation [43–46], which is critical because of the different therapeutic approaches for these diseases. We constructed a proteome-based workflow and identified DNAJC9 as a potential diagnostic marker of OABL. DNAJC9 is a heat shock protein family member that is a histone co-chaperone and a p53-target gene [47, 48]. In inflammation and OABL tissues, DNAJC9 was co-expressed with CD20 and predominantly localized in the nucleus. Our study demonstrates that nuclear staining of DNAJC9 is a promising pathology diagnostic biomarker of EMZL, which may provide important benefits in clinical practice.

## Conclusions

OABL is a rare subtype of non-Hodgkin lymphoma, and its molecular characteristic is poorly understood. We performed an integrated study to investigate the proteotranscriptome landscape of OABL. We found that alternative splicing may be the biological foundation for malignancy development. Furthermore, ADAR, a core SF, regulates the proliferation and Rho GTPase inhibitor sensitivity of NHL cell lines. OABL is characterized by high global protein-mRNA concordance, which is a novel recurrence-related characteristic. This study also identified the strong nuclear staining of DNAJC9 as a promising pathology diagnostic biomarker of EMZL. Our results provide insights into the biology of OABL and pave the way for clinical practice and further study of OABL.

## Supplementary Information

**Additional file 1.** Supplementary methods.

**Additional file 2: Figure S1.** Unsupervised hierarchical clustering heatmap of proteomic and transcriptome results. Highly variant transcripts/proteins (median absolute deviation top 1000) are included in the analyses.

**Additional file 3: Figure S2.** Poteotranscriptomics identify enrichment terms in OABLs. (A) The top 15 CO-UP and CO-DOWN gene sets identified by GSEA analyses are represented in a heatmap of median GSVA score across subgroups. (B) Bar plot of enrichment terms identified by no-coherent dysregulated protein-mRNA pairs. (C) Bubble plot of top 20 discordantly dysregulated gene sets identified by GSEA.

**Additional file 4: Figure S3.** Association between the global protein-mRNA concordance and disease characteristics. (A) Global protein-mRNA concordance is relatively associated with the high Ann Arbor Stage. (B) Kaplan-Meier survival analyses of progression-free survival, overall survival, recurrence-free survival, and local recurrence-free survival. (C) Boxplots show the association between global concordance and different recurrence patterns in OABL, SBL, EMZL, DLBCL, and other subtypes.

**Additional file 5: Figure S4.** Construction of the inflammation-OABL signature. (A) Elbow plot of k-means clustering. (B) Bar plot of top enrichment terms of the inflammation-OABL signature.

**Additional file 6: Figure S5.** Splicing factors play an important role in NHL. (A) The mRNA splicing gene set is significantly higher in all subtypes of OABL compared with the control group. (B) Bar plot or enriched domain of dysregulated proteins. (C) Genomic event of top 10 AASE correlated Splicing factors and regulators in DLBCL cohorts.

**Additional file 7: Figure S6.** Characteristics of ADAR knockdown NHL cell lines. (A) Representative western blots of ADAR expression in SU-DHL-4 and Raji cells with ADAR knockdown (shADAR) from three independent experiments. (B) Complete picture of the western blot. (C) Relative cell viability of SU-DHL-4 and Raji cells with ADAR knockdown (shADAR) and control treated by 100 μM HA-110 HCL for 72 hours. The cell viability is normalized by the corresponding cell treated by DMSO.

**Additional file 8: Table S1.** Patient characteristics.

**Additional file 9: Table S2.** Protein abundance of patients.

**Additional file 10: Table S3.** Transcriptomic FPKM value of patients.

**Additional file 11: Table S4.** Differentially expressed protein-mRNA pairs between OABLs and controls.

**Additional file 12: Table S5.** Differentially enriched gene sets identified by GSEA in transcriptomic and proteomic cohorts between OABLs and controls.

**Additional file 13: Table S6.** GSVA score of patients in the proteomic cohort.

**Additional file 14: Table S7.** Protein clusters results.

**Additional file 15: Table S8.** IncLevel of AASE of patients in the transcriptomic cohort.

**Additional file 16 **: **Table S9.** Annotated splicing factors and regulators.

**Additional file 17: Table S10.** Association of AASE with PFS in OABL calculated by cox regression analysis.

**Additional file 18: Table S11.** AASE analysis of ADAR knockdown/knockout/overexpression cancer cell lines.

## Data Availability

The proteomic data have been uploaded and available in the iProX database (https://www.iprox.org), the project ID is IPX0004253000. The RNA-seq data have been deposited and available in the Gene Expression Omnibus database under accession number GSE171059 and GSE199517 (https://www.ncbi.nlm.nih.gov/geo). FPKM values of RNA-seq data are obtained from The Cancer Genome Atlas (https://portal.gdc.cancer.gov/projects/). Fastq files of GEO series (GSE106874, GSE122168, GSE131658, GSE132287, GSE132288, GSE147487, GSE165282, GSE28040, GSE47997) are downloaded from SRA.

## Abbreviations

OAL: Ocular adnexal lymphoma
OABL: Ocular adnexal B-cell lymphoma
NHL: Non-Hodgkin lymphoma
EMZL: Extranodal marginal zone B-cell lymphoma
DLBCL: Diffuse large B-cell lymphoma
MCL: Mantle cell lymphoma
SLL: Small lymphocytic lymphoma
SBL: Small B-cell lymphoma
RLH: Reactive lymphoid hyperplasia
IOI: Idiopathic orbital inflammation
TMT: Tandem mass tags
MS: Mass spectrometry
RNA-seq: RNA sequencing
TCGA: The Cancer Genome Atlas
FDR: False discovery rate
PCA: Principle component analysis
GSEA: Gene set enrichment analysis
GSVA: Gene set variation analysis
FFPE: Formalin-fixed paraffin embedded
IHC: Immunohistochemistry
IF: Immunofluorescent
MFI: Mean fluorescent intensity
PFS: Progression-free survival
RFS: Recurrence-free survival
LRFS: Local recurrence-free survival
DRFS: Distant recurrence-free survival
OS: Overall survival
HR: Hazard ratio
CI: Confidence interval
MAD: Median absolute deviation
HVG: High variable gene
CO-DOWN: Concordantly downregulated
CO-UP: Concordantly upregulated
ECM: Extracellular matrix
ASE: Alternative splicing event
AASE: Aberrant alternative splicing event
SF: Splicing factor
A3: Alternative 3′ splice site
A5: Alternative 5′ splice site
MX: Mutually exclusive exon
RI: Retained intron
SE: Skipping exon
KD: Knockdown
IC50: Half maximal inhibitory concentration

## References

[1] Esmaeli B, Sniegowski M (2015). Orbital and ocular adnexal lymphoma. Orbital Tumors.

[2] Aronow ME, Hill BT, Singh AD (2014). Orbital and adnexal lymphoma. Clinical ophthalmic Oncology.

[3] Shimizu N, Oshitari T, Yotsukura J, Yokouchi H, Baba T, Yamamoto S (2021). Ten-year epidemiological study of ocular and orbital tumors in Chiba University Hospital. BMC Ophthalmol.

[4] Olsen TG, Holm F, Mikkelsen LH, Rasmussen PK, Coupland SE, Esmaeli B (2019). Orbital lymphoma-An international multicenter retrospective study. Am J Ophthalmol.

[5] Hsu CR, Chen YY, Yao M, Wei YH, Hsieh YT, Liao SL (2021). Orbital and ocular adnexal lymphoma: a review of epidemiology and prognostic factors in Taiwan. Eye (Lond).

[6] Holm F, Mikkelsen LH, Kamper P, Rasmussen PK, Larsen TS, Sjo LD (2021). Ocular adnexal lymphoma in Denmark: a nationwide study of 387 cases from 1980 to 2017. Br J Ophthalmol.

[7] Asakage M, Usui Y, Nezu N, Shimizu H, Tsubota K, Umazume K (2020). Comprehensive gene analysis of IgG4-related ophthalmic disease using RNA sequencing. J Clin Med.

[8] Ennishi D, Jiang A, Boyle M, Collinge B, Grande BM, Ben-Neriah S (2019). Double-hit gene expression signature defines a distinct subgroup of germinal center B-cell-like diffuse large B-cell lymphoma. J Clin Oncol.

[9] Loeffler-Wirth H, Kreuz M, Hopp L, Arakelyan A, Haake A, Cogliatti SB (2019). A modular transcriptome map of mature B cell lymphomas. Genome Med..

[10] Dunn J, Lenis VP, Hilton DA, Warta R, Herold-Mende C, Hanemann CO (2020). Integration and Comparison of Transcriptomic and Proteomic Data for Meningioma. Cancers (Basel).

[11] Tang W, Zhou M, Dorsey TH, Prieto DA, Wang XW, Ruppin E (2018). Integrated proteotranscriptomics of breast cancer reveals globally increased protein-mRNA concordance associated with subtypes and survival. Genome Med.

[12] Ule J, Blencowe BJ (2019). Alternative splicing regulatory networks: functions, mechanisms, and evolution. Mol Cell.

[13] Bonnal SC, Lopez-Oreja I, Valcarcel J (2020). Roles and mechanisms of alternative splicing in cancer - implications for care. Nat Rev Clin Oncol.

[14] Elenitoba-Johnson KSJ, Lim MS (2018). New insights into lymphoma pathogenesis. Annu Rev Pathol.

[15] Shen S, Park JW, Lu ZX, Lin L, Henry MD, Wu YN (2014). rMATS: robust and flexible detection of differential alternative splicing from replicate RNA-Seq data. Proc Natl Acad Sci U S A.

[16] Giulietti M, Piva F, D'Antonio M, D'Onorio De Meo P, Paoletti D, Castrignano T (2013). SpliceAid-F: a database of human splicing factors and their RNA-binding sites. Nucleic Acids Res.

[17] Van Nostrand EL, Freese P, Pratt GA, Wang X, Wei X, Xiao R (2020). A large-scale binding and functional map of human RNA-binding proteins. Nature..

[18] Ritchie ME, Phipson B, Wu D, Hu Y, Law CW, Shi W (2015). Limma powers differential expression analyses for RNA-sequencing and microarray studies. Nucleic Acids Res.

[19] Zhou Y, Zhou B, Pache L, Chang M, Khodabakhshi AH, Tanaseichuk O (2019). Metascape provides a biologist-oriented resource for the analysis of systems-level datasets. Nat Commun.

[20] Mootha VK, Lindgren CM, Eriksson KF, Subramanian A, Sihag S, Lehar J (2003). PGC-1alpha-responsive genes involved in oxidative phosphorylation are coordinately downregulated in human diabetes. Nat Genet.

[21] Subramanian A, Tamayo P, Mootha VK, Mukherjee S, Ebert BL, Gillette MA (2005). Gene set enrichment analysis: a knowledge-based approach for interpreting genome-wide expression profiles. Proc Natl Acad Sci U S A.

[22] Hanzelmann S, Castelo R, Guinney J (2013). GSVA: gene set variation analysis for microarray and RNA-seq data. BMC Bioinformatics.

[23] Gentleman RC, Carey VJ, Bates DM, Bolstad B, Dettling M, Dudoit S (2004). Bioconductor: open software development for computational biology and bioinformatics. Genome Biol.

[24] Maier T, Guell M, Serrano L (2009). Correlation of mRNA and protein in complex biological samples. FEBS Lett.

[25] Viatour P, Merville MP, Bours V, Chariot A (2005). Phosphorylation of NF-kappaB and IkappaB proteins: implications in cancer and inflammation. Trends Biochem Sci.

[26] Kennedy R, Klein U (2018). Aberrant activation of NF-kappaB Signalling in aggressive lymphoid malignancies. Cells..

[27] Smedby KE, Hjalgrim H, Askling J, Chang ET, Gregersen H, Porwit-MacDonald A (2006). Autoimmune and chronic inflammatory disorders and risk of non-Hodgkin lymphoma by subtype. J Natl Cancer Inst.

[28] Smedby KE, Ponzoni M (2017). The aetiology of B-cell lymphoid malignancies with a focus on chronic inflammation and infections. J Intern Med.

[29] Pradella D, Naro C, Sette C, Ghigna C (2017). EMT and stemness: flexible processes tuned by alternative splicing in development and cancer progression. Mol Cancer.

[30] Siddaway R, Milos S, Vadivel AKA, Dobson THW, Swaminathan J, Ryall S (2022). Splicing is an alternate oncogenic pathway activation mechanism in glioma. Nat Commun.

[31] Cerami E, Gao J, Dogrusoz U, Gross BE, Sumer SO, Aksoy BA (2012). The cBio cancer genomics portal: an open platform for exploring multidimensional cancer genomics data. Cancer Discov.

[32] Tang SJ, Shen H, An O, Hong H, Li J, Song Y (2020). Cis- and trans-regulations of pre-mRNA splicing by RNA editing enzymes influence cancer development. Nat Commun.

[33] Voena C, Chiarle R (2019). RHO family GTPases in the biology of lymphoma. Cells..

[34] Crosas-Molist E, Samain R, Kohlhammer L, Orgaz JL, George SL, Maiques O (2022). Rho GTPase signaling in cancer progression and dissemination. Physiol Rev.

[35] Olsen TG, Heegaard S (2019). Orbital lymphoma. Surv Ophthalmol.

[36] Anastasiadou E, Jacob LS, Slack FJ (2018). Non-coding RNA networks in cancer. Nat Rev Cancer.

[37] Zhou Z, Lv J, Yu H, Han J, Yang X, Feng D (2020). Mechanism of RNA modification N6-methyladenosine in human cancer. Mol Cancer.

[38] Ostergaard Poulsen M, Krogh Jorgensen L, Sorensen S, Falgreen S, Stove Bodker JS, Bach Laursen M (2016). Alternative pre-mRNA splicing leads to potential biomarkers in diffuse large B-cell lymphoma - a systematic review. Dan Med J.

[39] Ishizuka JJ, Manguso RT, Cheruiyot CK, Bi K, Panda A, Iracheta-Vellve A (2019). Loss of ADAR1 in tumours overcomes resistance to immune checkpoint blockade. Nature..

[40] Herbert A (2019). ADAR and immune silencing in Cancer. Trends Cancer.

[41] Lv X, Gu C, Guo S (2020). Activation of BDNF-AS/ADAR/p53 positive feedback loop inhibits glioblastoma cell proliferation. Neurochem Res.

[42] Wu Z, Zhou J, Zhang X, Zhang Z, Xie Y, Liu JB (2021). Reprogramming of the esophageal squamous carcinoma epigenome by SOX2 promotes ADAR1 dependence. Nat Genet.

[43] Sun B, Song L, Wang X, Li J, Xian J, Wang F (2017). Lymphoma and inflammation in the orbit: diagnostic performance with diffusion-weighted imaging and dynamic contrast-enhanced MRI. J Magn Reson Imaging.

[44] Marino M, Ionni I, Lanzolla G, Sframeli A, Latrofa F, Rocchi R (2020). Orbital diseases mimicking graves’ orbitopathy: a long-standing challenge in differential diagnosis. J Endocrinol Investig.

[45] Raderer M, Kiesewetter B, Ferreri AJ (2016). Clinicopathologic characteristics and treatment of marginal zone lymphoma of mucosa-associated lymphoid tissue (MALT lymphoma). CA Cancer J Clin.

[46] Kalogeropoulos D, Papoudou-Bai A, Kanavaros P, Kalogeropoulos C (2018). Ocular adnexal marginal zone lymphoma of mucosa-associated lymphoid tissue. Clin Exp Med.

[47] Mandriani B, Castellana S, Rinaldi C, Manzoni M, Venuto S, Rodriguez-Aznar E (2016). Identification of p53-target genes in Danio rerio. Sci Rep.

[48] Hammond CM, Bao H, Hendriks IA, Carraro M, Garcia-Nieto A, Liu Y (2021). DNAJC9 integrates heat shock molecular chaperones into the histone chaperone network. Mol Cell.

