# Supplementary material for: Proteotranscriptomics of ocular adnexal B-cell lymphoma reveals an oncogenic role of alternative splicing and identifies a diagnostic marker
Source: J Exp Clin Cancer Res. 2022 Jul 30;41:234. doi: 10.1186/s13046-022-02445-8 (PMC9338531; doi:10.1186/s13046-022-02445-8)
Supplement: Supplementary file 1 — Additional file 1. Supplementary methods. [file 13046_2022_2445_MOESM1_ESM.docx]

**Supplementary Methods**

**Quantitative proteomic sequencing**

Specimens were lysed using protein extraction buffer (8 M urea,0.1% SDS) containing additional 1 mM phenylmethylsulfonyl fluoride (Beyotime Biotechnology, China) and protease inhibitor cocktail (Roche, USA).

Tandem mass tags TMTpro (Pierce, USA) with different reporter ions (126-131 Da) were applied as isobaric tags for relative quantification and TMT labeling was performed according to the manufacturer’s instructions. The three labeled peptide aliquots were combined for subsequent fractionation. A total of 40 fractions were collected which were then concatenated to 20 fractions, vacuum dried and stored at -80℃ until further analysis.

The LC-MS/MS analysis was carried out by using Q Exactive mass spectrometer (Thermo Scientific, USA). Mass spectrometry analysis was performed in a data dependent manner with full scans (350-1,600 m/z) acquired using an Orbitrap mass analyzer at a mass resolution of 70,000 at 400 m/z in Q Exactive. Twenty most intense precursor ions from a survey scan were selected for MS/MS from each duty cycle and detected at a mass resolution of 35,000 at m/z of 400 in Orbitrap analyzer. All the tandem mass spectra were produced by higher-energy collision dissociation (HCD) method.

Proteome discoverer software (version 1.4) (Thermo Scientific, USA) was used to perform database searching against the NCBI’s RefSeq human protein sequence database using the Sequest algorithms. Following criteria were applied: precursor mass tolerance of 15 ppm, fragment mass tolerance of 20 mmu. Trypsin was specified as digesting enzyme and 2 missed cleavages are allowed. Cysteine carbamidomethylation and TMT modifications (N-terminus and lysine residues) were defined as fixed modifications and methionine oxidation was variable modifications. The results were filtered using following settings: only high confident peptides with a global FDR<1% based on a target-decoy approach were included in the results. In the TMT quantitation workflow, the most confident centroid method was used with an integration window of 20 ppm. For protein quantitation, only unique peptides were used to quantify abundance.

**Construction of dysregulated protein pattern by t-test**

Previously curated protein abundance matrix (Supplementary Table S2) was used to perform the t-test of protein abundance between every two subgroups. In total, three times of t-tests were performed (normal vs inflammation; inflammation vs OABL; normal vs. OABL).

We hypothesized 9 dysregulation patterns (Fig. 3F). Proteins in cluster 1 were failed to show significant dysregulation across groups. Proteins in cluster 2u/d were significantly up/downregulated in all three analyses. In cluster 3u/d, proteins were stable between inflammation and OABL, but significantly up/downregulated in both when compared with normal. In cluster 4u/d, proteins were stable in control groups, but significantly up/downregulated in OABL compared with any of inflammation or normal. Other proteins significantly dysregulated in inflammation group compared to normal were included in 5u/d.

**Correlation score calculation**

The correlation score is calculated as the following description.

Correlation score = a1X10-2+ a2X10-1+ a3X100+ a4X101+ a5X102

a1 = count of AASEs with |0≤r<0.2|

a2 = count of AASEs with |0.2≤r<0.4|

a3 = count of AASEs with |0.4≤r<0.6|

a4 = count of AASEs with |0.6≤r<0.8|

a5 = count of AASEs with |0.8≤r<1.0|

**Analyzing alternative splicing in GEO series**

Fastq files of ADAR KD/knockout/overexpression studies in cancer cells (GSE106874, GSE122168, GSE131658, GSE132287, GSE132288, GSE147487, GSE165282, GSE28040, GSE47997) were download from SRA.

AASEs were analyzed with rMATS as previously described in Materials and Methods. All AASEs were merged in one table and labeled as “splicing type | Ensembl number”. The Frequency of detection was calculated. Labeled AASEs detected in at least two groups of cell line was identified as recurrent AASEs. Genes of these AASEs were defined as “ADAR regulated AASE affected gene” and put into the enrichment analysis.

**Data availability**

FPKM values of RNA sequnecing data were obtained from The Cancer Genome Atlas (<https://portal.gdc.cancer.gov/projects/>) to evaluate the correlation between MKI67 and ADAR abundance in pan-cancer. Fastq files of GEO series (GSE106874, GSE122168, GSE131658, GSE132287, GSE132288, GSE147487, GSE165282, GSE28040, GSE47997) were downloaded from SRA to evaluate the core regulator role of ADAR.
