# Supplementary figures and images for: Proteotranscriptomics of ocular adnexal B-cell lymphoma reveals an oncogenic role of alternative splicing and identifies a diagnostic marker
Source: J Exp Clin Cancer Res. 2022 Jul 30;41:234. doi: 10.1186/s13046-022-02445-8 (PMC9338531; doi:10.1186/s13046-022-02445-8)

## Figure S1

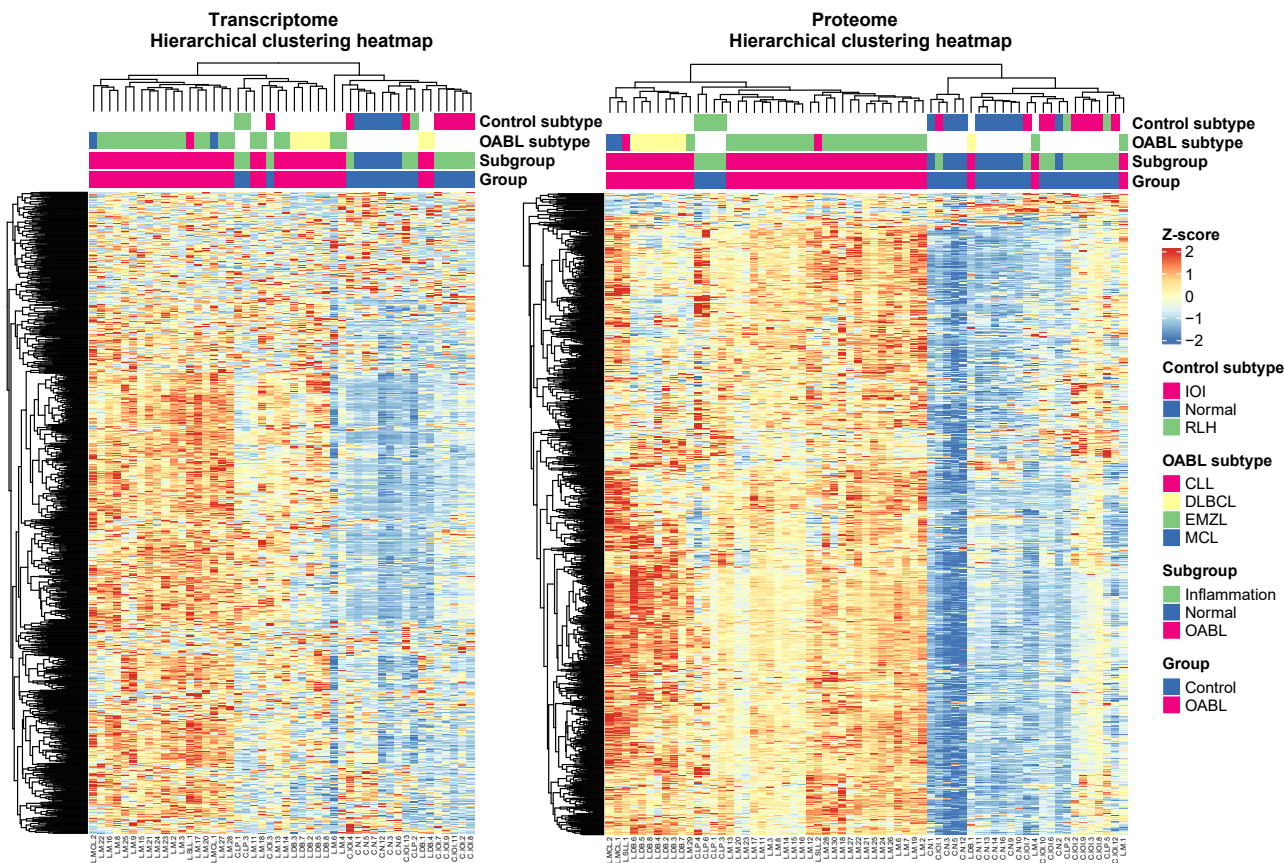

Supplement: Supplementary file 2 — Additional file 2: Figure S1. Unsupervised hierarchical clustering heatmap of proteomic and transcriptome results. Highly variant transcripts/proteins (median absolute deviation top 1000) are included in the analyses. [file 13046_2022_2445_MOESM2_ESM.pdf]

Figure S2

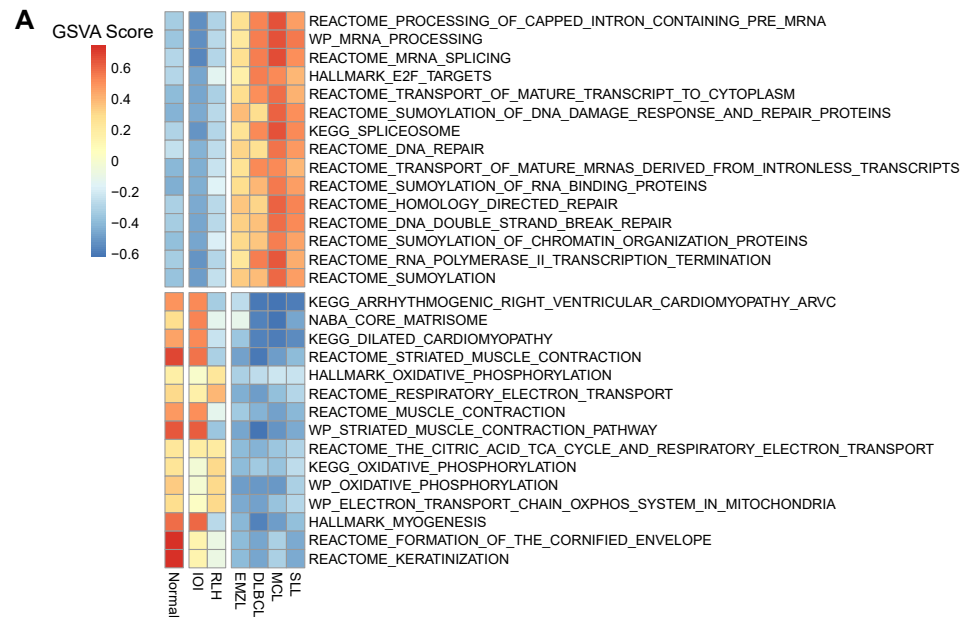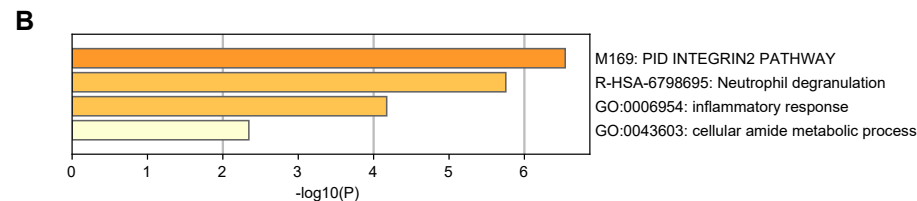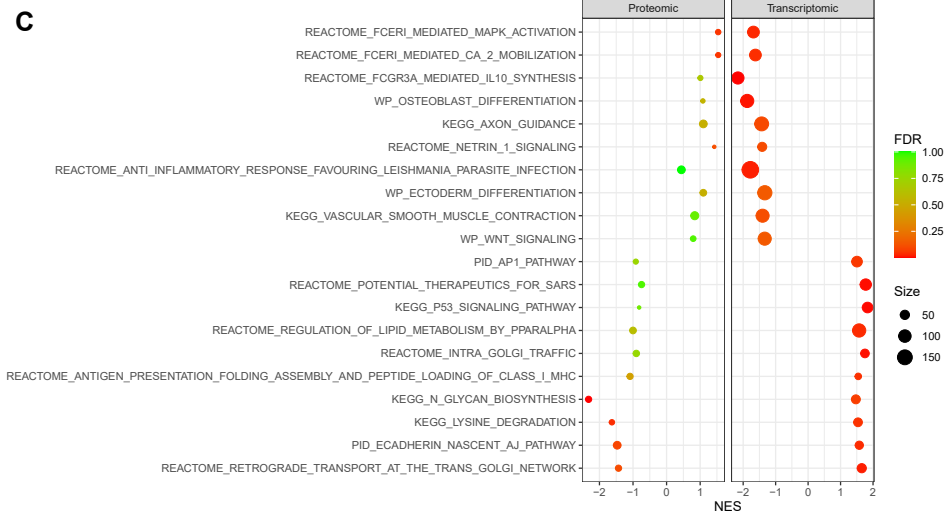

Supplement: Supplementary file 3 — Additional file 3: Figure S2. Poteotranscriptomics identify enrichment terms in OABLs. (A) The top 15 CO-UP and CO-DOWN gene sets identified by GSEA analyses are represented in a heatmap of median GSVA score across subgroups. (B) Bar plot of enrichment terms identified by no-coherent dysregulated protein-mRNA pairs. (C) Bubble plot of top 20 discordantly dysregulated gene sets identified by GSEA. [file 13046_2022_2445_MOESM3_ESM.pdf]

**Figure S3****A**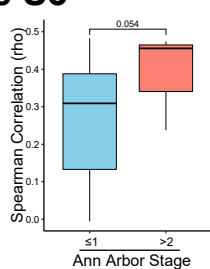**B**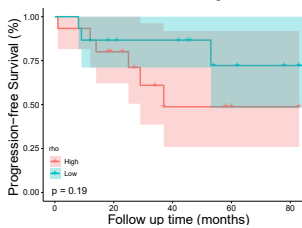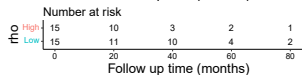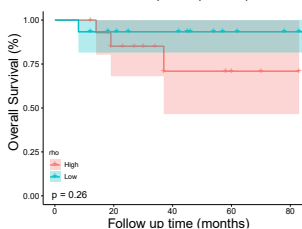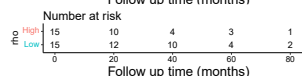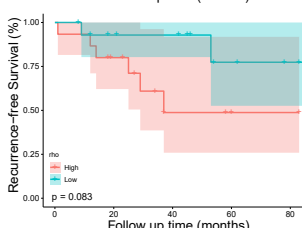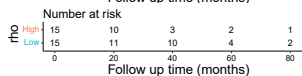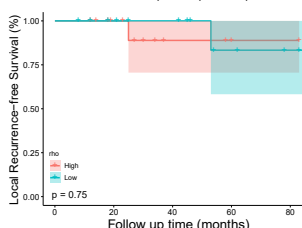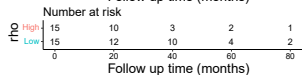**C**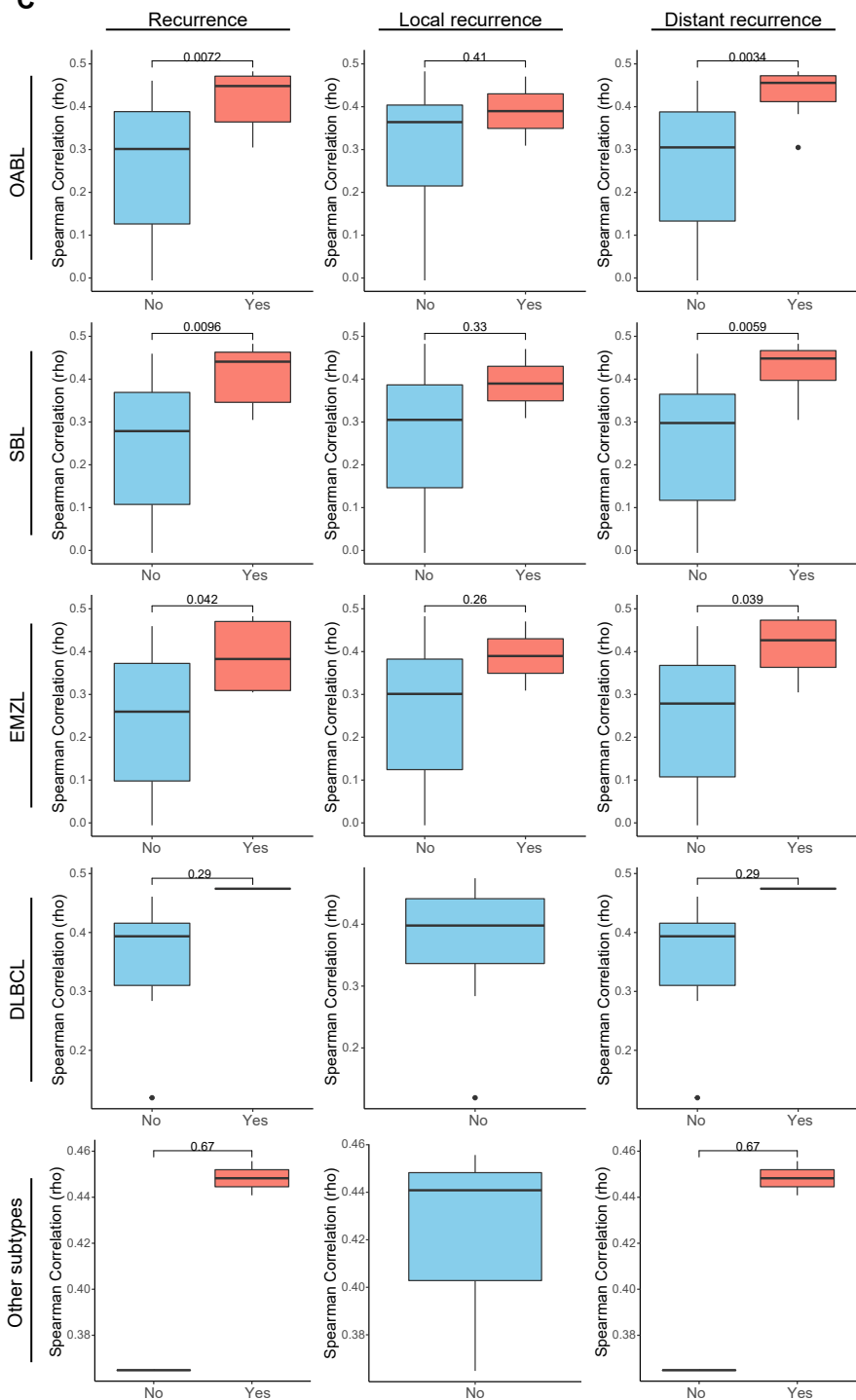

Supplement: Supplementary file 4 — Additional file 4: Figure S3. Association between the global protein-mRNA concordance and disease characteristics. (A) Global protein-mRNA concordance is relatively associated with the high Ann Arbor Stage. (B) Kaplan-Meier survival analyses of progression-free survival, overall survival, recurrence-free survival, and local recurrence-free survival. (C) Boxplots show the association between global concordance and different recurrence patterns in OABL, SBL, EMZL, DLBCL, and other subtypes. [file 13046_2022_2445_MOESM4_ESM.pdf]

Figure S4

A

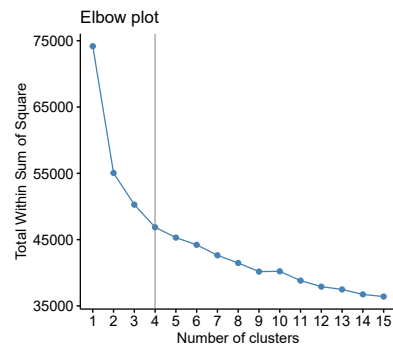

B

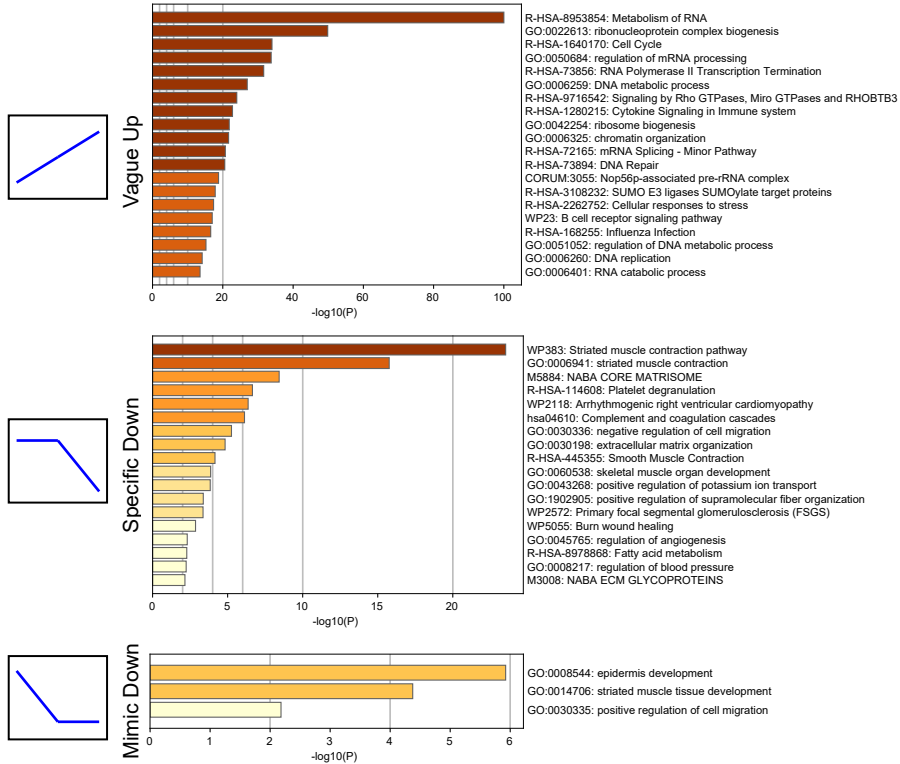

Supplement: Supplementary file 5 — Additional file 5: Figure S4. Construction of the inflammation-OABL signature. (A) Elbow plot of k-means clustering. (B) Bar plot of top enrichment terms of the inflammation-OABL signature. [file 13046_2022_2445_MOESM5_ESM.pdf]

Figure S5

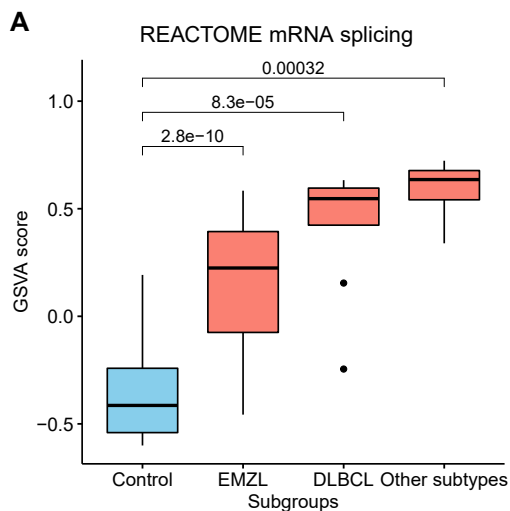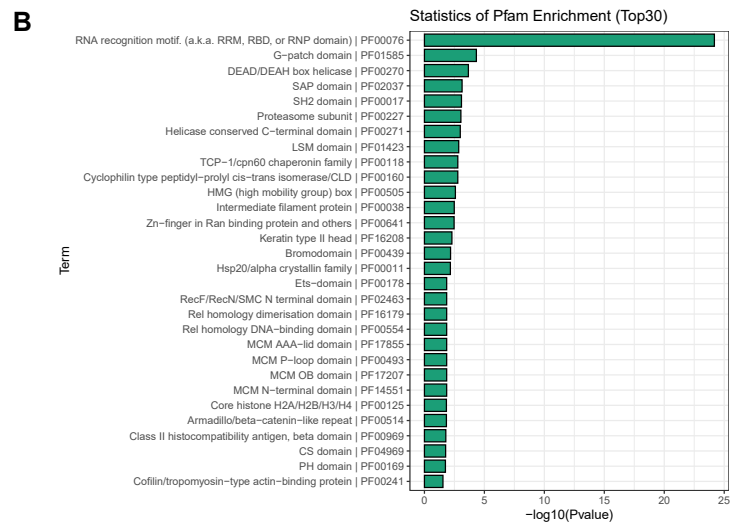

**B**

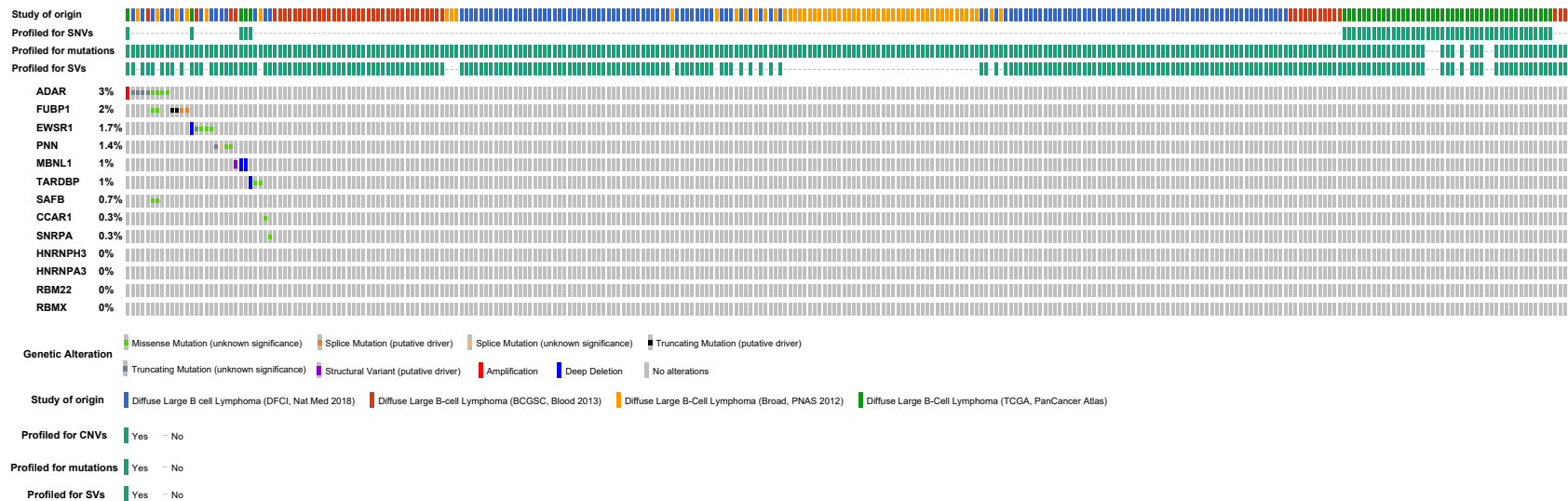

Supplement: Supplementary file 6 — Additional file 6: Figure S5. Splicing factors play an important role in NHL. (A) The mRNA splicing gene set is significantly higher in all subtypes of OABL compared with the control group. (B) Bar plot or enriched domain of dysregulated proteins. (C) Genomic event of top 10 AASE correlated Splicing factors and regulators in DLBCL cohorts. [file 13046_2022_2445_MOESM6_ESM.pdf]

**Figure S6**

**A**

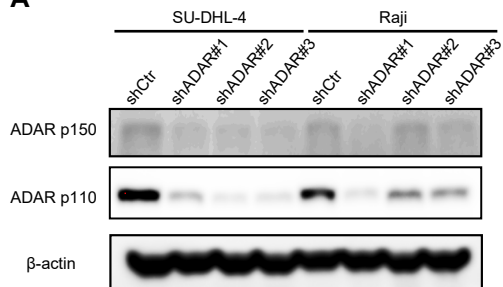

**B**

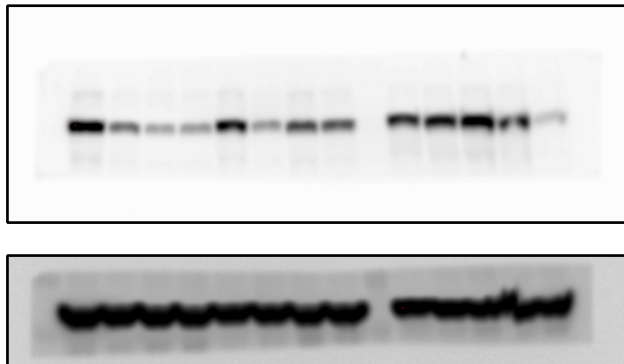

**C**

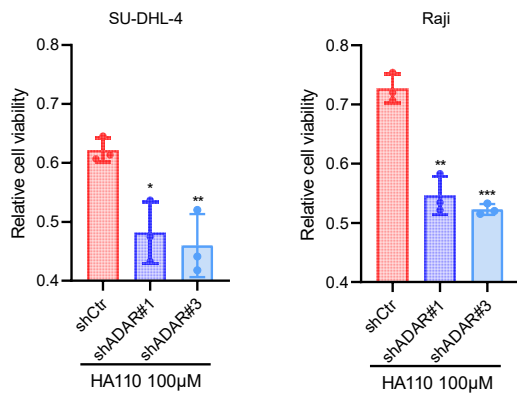

Supplement: Supplementary file 7 — Additional file 7: Figure S6. Characteristics of ADAR knockdown NHL cell lines. (A) Representative western blots of ADAR expression in SU-DHL-4 and Raji cells with ADAR knockdown (shADAR) from three independent experiments. (B) Complete picture of the western blot. (C) Relative cell viability of SU-DHL-4 and Raji cells with ADAR knockdown (shADAR) and control treated by 100 μM HA-110 HCL for 72 hours. The cell viability is normalized by the corresponding cell treated by DMSO. [file 13046_2022_2445_MOESM7_ESM.pdf]
